# Supplementary material for: Pyruvate Dehydrogenase Kinase 1 inhibition mediated oxidative phosphorylation enhancement in cartilage promotes osteoarthritis progression
Source: BMC Musculoskelet Disord. 2023 Jul 20;24:597. doi: 10.1186/s12891-023-06585-6 (PMC10357736; doi:10.1186/s12891-023-06585-6)
Supplement: Supplementary file 1 — Additional file 1. [file 12891_2023_6585_MOESM1_ESM.zip › MF.Gsea.1653627316242/GOMF_PROTEIN_TYROSINE_SERINE_THREONINE_PHOSPHATASE_ACTIVITY.html]

Details for gene set GOMF\_PROTEIN\_TYROSINE\_SERINE\_THREONINE\_PHOSPHATASE\_ACTIVITY[GSEA]

|  || Dataset | 55457\_collapsed\_to\_symbols.cls.cls #OA\_versus\_normal.cls.cls #OA\_versus\_normal\_repos |
| Phenotype | cls.cls#OA\_versus\_normal\_repos |
| Upregulated in class | normal |
| GeneSet | GOMF\_PROTEIN\_TYROSINE\_SERINE\_THREONINE\_PHOSPHATASE\_ACTIVITY |
| Enrichment Score (ES) | -0.43200448 |
| Normalized Enrichment Score (NES) | -1.4843452 |
| Nominal p-value | 0.028380634 |
| FDR q-value | 0.19525473 |
| FWER p-Value | 1.0 |
Table: GSEA Results Summary

  

Fig 1: Enrichment plot: GOMF\_PROTEIN\_TYROSINE\_SERINE\_THREONINE\_PHOSPHATASE\_ACTIVITY      
 Profile of the Running ES Score & Positions of GeneSet Members on the Rank Ordered List

  

| SYMBOL | TITLE | RANK IN GENE LIST | RANK METRIC SCORE | RUNNING ES | CORE ENRICHMENT || 1 | STYXL1 | serine/threonine/tyrosine interacting like 1 [Source:HGNC Symbol;Acc:HGNC:18165] | 244 | 0.787 | 0.0436 | No |
| 2 | DUSP22 | dual specificity phosphatase 22 [Source:HGNC Symbol;Acc:HGNC:16077] | 998 | 0.518 | 0.0263 | No |
| 3 | CDKN3 | cyclin dependent kinase inhibitor 3 [Source:HGNC Symbol;Acc:HGNC:1791] | 1422 | 0.440 | 0.0284 | No |
| 4 | DUSP12 | dual specificity phosphatase 12 [Source:HGNC Symbol;Acc:HGNC:3067] | 1802 | 0.384 | 0.0295 | No |
| 5 | DUSP14 | dual specificity phosphatase 14 [Source:HGNC Symbol;Acc:HGNC:17007] | 1921 | 0.370 | 0.0498 | No |
| 6 | DUSP11 | dual specificity phosphatase 11 [Source:HGNC Symbol;Acc:HGNC:3066] | 3135 | 0.237 | -0.0257 | No |
| 7 | SSH3 | slingshot protein phosphatase 3 [Source:HGNC Symbol;Acc:HGNC:30581] | 3707 | 0.185 | -0.0553 | No |
| 8 | RNGTT | RNA guanylyltransferase and 5'-phosphatase [Source:HGNC Symbol;Acc:HGNC:10073] | 4737 | 0.092 | -0.1280 | No |
| 9 | DUSP3 | dual specificity phosphatase 3 [Source:HGNC Symbol;Acc:HGNC:3069] | 4857 | 0.083 | -0.1306 | No |
| 10 | CDC14B | cell division cycle 14B [Source:HGNC Symbol;Acc:HGNC:1719] | 5099 | 0.064 | -0.1443 | No |
| 11 | DUSP13 | dual specificity phosphatase 13 [Source:HGNC Symbol;Acc:HGNC:19681] | 6110 | -0.013 | -0.2217 | No |
| 12 | MTMR2 | myotubularin related protein 2 [Source:HGNC Symbol;Acc:HGNC:7450] | 6189 | -0.019 | -0.2262 | No |
| 13 | PPM1F | "protein phosphatase, Mg2+/Mn2+ dependent 1F [Source:HGNC Symbol;Acc:HGNC:19388]" | 6191 | -0.020 | -0.2247 | No |
| 14 | DUSP7 | dual specificity phosphatase 7 [Source:HGNC Symbol;Acc:HGNC:3073] | 6966 | -0.077 | -0.2787 | No |
| 15 | DUSP10 | dual specificity phosphatase 10 [Source:HGNC Symbol;Acc:HGNC:3065] | 7775 | -0.140 | -0.3304 | No |
| 16 | PTP4A3 | protein tyrosine phosphatase 4A3 [Source:HGNC Symbol;Acc:HGNC:9636] | 7984 | -0.154 | -0.3343 | No |
| 17 | DUSP21 | dual specificity phosphatase 21 [Source:HGNC Symbol;Acc:HGNC:20476] | 8323 | -0.180 | -0.3463 | No |
| 18 | DUSP26 | dual specificity phosphatase 26 [Source:HGNC Symbol;Acc:HGNC:28161] | 8424 | -0.187 | -0.3392 | No |
| 19 | PTP4A2 | protein tyrosine phosphatase 4A2 [Source:HGNC Symbol;Acc:HGNC:9635] | 8806 | -0.217 | -0.3515 | No |
| 20 | PTP4A1 | protein tyrosine phosphatase 4A1 [Source:HGNC Symbol;Acc:HGNC:9634] | 8874 | -0.223 | -0.3390 | No |
| 21 | SSH1 | slingshot protein phosphatase 1 [Source:HGNC Symbol;Acc:HGNC:30579] | 9748 | -0.296 | -0.3833 | No |
| 22 | TPTE | transmembrane phosphatase with tensin homology [Source:HGNC Symbol;Acc:HGNC:12023] | 10002 | -0.321 | -0.3774 | No |
| 23 | SBF1 | SET binding factor 1 [Source:HGNC Symbol;Acc:HGNC:10542] | 10406 | -0.361 | -0.3800 | No |
| 24 | MTMR3 | myotubularin related protein 3 [Source:HGNC Symbol;Acc:HGNC:7451] | 10751 | -0.399 | -0.3750 | No |
| 25 | DUSP9 | dual specificity phosphatase 9 [Source:HGNC Symbol;Acc:HGNC:3076] | 11486 | -0.500 | -0.3923 | Yes |
| 26 | DUSP2 | dual specificity phosphatase 2 [Source:HGNC Symbol;Acc:HGNC:3068] | 11768 | -0.544 | -0.3708 | Yes |
| 27 | PTEN | phosphatase and tensin homolog [Source:HGNC Symbol;Acc:HGNC:9588] | 11836 | -0.556 | -0.3318 | Yes |
| 28 | DUSP4 | dual specificity phosphatase 4 [Source:HGNC Symbol;Acc:HGNC:3070] | 12068 | -0.612 | -0.3011 | Yes |
| 29 | DUSP8 | dual specificity phosphatase 8 [Source:HGNC Symbol;Acc:HGNC:3074] | 12303 | -0.678 | -0.2653 | Yes |
| 30 | DUSP6 | dual specificity phosphatase 6 [Source:HGNC Symbol;Acc:HGNC:3072] | 12644 | -0.821 | -0.2264 | Yes |
| 31 | DUSP5 | dual specificity phosphatase 5 [Source:HGNC Symbol;Acc:HGNC:3071] | 12721 | -0.882 | -0.1621 | Yes |
| 32 | CDC14A | cell division cycle 14A [Source:HGNC Symbol;Acc:HGNC:1718] | 12823 | -1.009 | -0.0897 | Yes |
| 33 | DUSP1 | dual specificity phosphatase 1 [Source:HGNC Symbol;Acc:HGNC:3064] | 12884 | -1.202 | 0.0012 | Yes |
Table: GSEA details [plain text format]

  

Fig 2: GOMF\_PROTEIN\_TYROSINE\_SERINE\_THREONINE\_PHOSPHATASE\_ACTIVITY      
 Blue-Pink O' Gram in the Space of the Analyzed GeneSet

  

Fig 3: GOMF\_PROTEIN\_TYROSINE\_SERINE\_THREONINE\_PHOSPHATASE\_ACTIVITY: Random ES distribution      
 Gene set null distribution of ES for **GOMF\_PROTEIN\_TYROSINE\_SERINE\_THREONINE\_PHOSPHATASE\_ACTIVITY**
